# Echocardiography-based AI detection of regional wall motion abnormalities and quantification of cardiac function in myocardial infarction

**DOI:** 10.3389/fcvm.2022.903660

**Published:** 2022-08-22

**Authors:** Xixiang Lin, Feifei Yang, Yixin Chen, Xiaotian Chen, Wenjun Wang, Xu Chen, Qiushuang Wang, Liwei Zhang, Huayuan Guo, Bohan Liu, Liheng Yu, Haitao Pu, Peifang Zhang, Zhenzhou Wu, Xin Li, Daniel Burkhoff, Kunlun He

**Affiliations:** ^1^Medical Big Data Center, Chinese PLA General Hospital, Beijing, China; ^2^Medical School of Chinese PLA, Beijing, China; ^3^BioMind Technology, Beijing, China; ^4^Fourth Medical Center of PLA General Hospital, Beijing, China; ^5^Sixth Medical Center of PLA General Hospital, Beijing, China; ^6^Cardiovascular Research Foundation, New York, NY, United States

**Keywords:** artificial intelligence - AI, myocardial infarction, echocardiography, deep learning, bedside ultrasound

## Abstract

**Objective:**

To compare the performance of a newly developed deep learning (DL) framework for automatic detection of regional wall motion abnormalities (RWMAs) for patients presenting with the suspicion of myocardial infarction from echocardiograms obtained with portable bedside equipment versus standard equipment.

**Background:**

Bedside echocardiography is increasingly used by emergency department setting for rapid triage of patients presenting with chest pain. However, compared to images obtained with standard equipment, lower image quality from bedside equipment can lead to improper diagnosis. To overcome these limitations, we developed an automatic workflow to process echocardiograms, including view selection, segmentation, detection of RWMAs and quantification of cardiac function that was trained and validated on image obtained from bedside and standard equipment.

**Methods:**

We collected 4,142 examinations from one hospital as training and internal testing dataset and 2,811 examinations from other hospital as the external test dataset. For data pre-processing, we adopted DL model to automatically recognize three apical views and segment the left ventricle. Detection of RWMAs was achieved with 3D convolutional neural networks (CNN). Finally, DL model automatically measured the size of cardiac chambers and left ventricular ejection fraction.

**Results:**

The view selection model identified the three apical views with an average accuracy of 96%. The segmentation model provided good agreement with manual segmentation, achieving an average Dice of 0.89. In the internal test dataset, the model detected RWMAs with AUC of 0.91 and 0.88 respectively for standard and bedside ultrasound. In the external test dataset, the AUC were 0.90 and 0.85. The automatic cardiac function measurements agreed with echocardiographic report values (e. g., mean bias is 4% for left ventricular ejection fraction).

**Conclusion:**

We present a fully automated echocardiography pipeline applicable to both standard and bedside ultrasound with various functions, including view selection, quality control, segmentation, detection of the region of wall motion abnormalities and quantification of cardiac function.

## Introduction

Myocardial infarction (MI) is the most severe manifestation of coronary heart disease, resulting in disability or sudden cardiac death. According to Report on Cardiovascular Health and Diseases in China 2021, AMI mortality increased by a factor 3.5 in rural areas and by a factor of 2.66 in urban areas from 2002 to 2019. In 2019, AMI mortality was 0.08% in rural areas and 0.06% in urban areas (1). Recent studies show that there is significant variability in the care and outcomes of MI patients in hospitals with different levels of care (2). Rapid diagnosis and prompt reperfusion treatment are of primary importance to reduce mortality from MI.

With the advantages of easy availability, low cost, fast performance and safety, transthoracic echocardiography (especially bedside ultrasound) is the most commonly used non-invasive imaging tool for detecting regional wall motion abnormalities (RWMAs) and providing information on short- and long-term outcomes after acute myocardial infarction (AMI) (3–5). The American College of Cardiology/American Heart Association and the European Heart Association guidelines give a Class I recommendation for using transthoracic echocardiography to detect RWMAs in chest pain patients presenting to the emergency ward without delaying angiography (6, 7).

However, accurate recognition of RWMAs by echocardiography requires highly trained and experienced physicians which are in short supply and typically not available around the clock in many hospitals. Furthermore, visual diagnosis of RWMAs often varies amongst doctors with various level of expertise (8). Therefore, an effective solution for efficient, accurate and objective diagnosis of RWMAs is needed.

Deep learning (DL) models have strong data processing capabilities and have been used for automated interpretation of images obtained from various modalities. Related to echocardiography, DL models can perform a variety of analyses, such as image quality assessment, view classification, boundary segmentation, and disease diagnosis (9–14). With the help of DL, tedious and time-consuming tasks like segmentation and quantification of different parameters can be performed quickly and precisely, saving increasingly scarce human resources (11, 13, 14).

Recently, tremendous advances have been made in DL models for the detection of RWMAs (15, 16). However, these studies applied relatively strict, up front image quality criteria such that ∼40% of studies were excluded from analysis, indicating that those models may not be practical for widespread use. Furthermore, in those studies, standard echocardiographic equipment was used which, in general, produce higher quality images than newer, portable bedside ultrasound equipment. With the advantages of portability and availability, bedside ultrasound is becoming increasingly applied in emergency rooms and intensive care units for specific applications, such as real-time assessment of cardiac function and RWMA in patients presenting with chest pain syndromes. Use of DL models to analyze images from these machines has not been specifically explored in prior studies (9, 10).

We developed a novel DL model to analyze echocardiographic videos to detect RWMAs and standard indexes of cardiac size and function from three standardized apical views. In contrast to prior studies, the structure of our model and the training dataset were geared toward analysis of images from bedside echocardiograms while fully retaining the ability to analyze images from standard equipment. Accordingly, the main purpose of this study was to compare the accuracy of this model for analyzing videos obtained from bedside ultrasound to those of standard equipment.

## Materials and methods

### Study population

**FIGURE 1:**
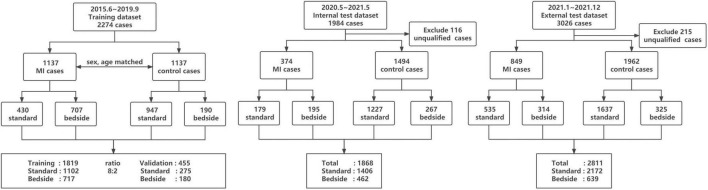
Summary of number of echocardiograms used in this study.

The methods used in the design, implementation, and reporting of this study were consistent with the recently published PRIME (Proposed Requirements for Cardiovascular Imaging Related Machine Learning Evaluation) checklist (17), which was provided in the Supplementary Appendix. We retrospectively accessed a total of 2,274 transthoracic echocardiographic examinations obtained between May 2015 and September 2019 from the Fourth Medical Center of Chinese PLA General Hospital as our training and validation dataset (ratio 8:2). MI and control cases were matched for age and sex. We then prospectively collected 1,868 examinations between May 2020 and May 2021 from the same hospital as an internal test dataset. For the external test dataset, we collected 3,026 examinations between Jan 2021 and Dec 2021 from the Second Affiliated Hospital of Shandong University of Traditional Chinese Medicine. Training and testing datasets each included echocardiographic studies from standard and bedside echocardiographic equipment as detailed in the Figure 1. The diagnosis of acute or prior MI were based on information from the electronic medical records (including echocardiographic report, blood tests, ECGs and angiograms). The presence and extent of RWMAs were extracted from echocardiographic reports generated by experienced sonographers and the echocardiograms were reviewed a second time by an experienced cardiologists who authorized the final diagnoses.

### Echocardiography

Each echocardiographic examination was acquired through standard methods. Videos from three standard apical views were include in this study: apical 4-chamber (A4C), apical 2-chamber (A2C), and apical long axis (ALX). Images were acquired from a diverse array of standard echocardiography machine manufacturers including Phillips EPIQ 7C and iE-elite with S5-1 and X5-1 transducers (Phillips, Andover, MA, United States), and Vivid E95 (General Electric, Fairfield, CT, United States) and portable bedside machines including Philips CX50 and Mindray M9cv with transducer SP5-1s (Mindray, Shenzhen, Guangdong, China). All images were stored with a standard Digital Imaging and Communication in Medicine (DICOM) format according to the instructions from each manufacturer.

### View selection and quality control

We labeled 33,404 images to develop a method to classify 29 standard views and then selected the three apical views required for the subsequent analysis. View selection was performed using a Xception Net neural network model according to methods that were previously described (18).

An automated algorithm was developed to assess image quality and exclude images whose quality were insufficient for analysis. Expert echocardiographers manually labeled 2,837 A4C, 1,880 A2C, and 1,910 ALX images as qualified or unqualified. These labeled images were then used to build the AI model. Examples of qualified and unqualified images as assigned by the model are shown in Supplementary Figure 1. As seen in these examples, the contour of left ventricle was ambiguous so that the endocardial or epicardial border was rarely identified in unqualified views. Subsequently, images automatically classified as unqualified were excluded from analysis.

### Segmentation

**FIGURE 2:**
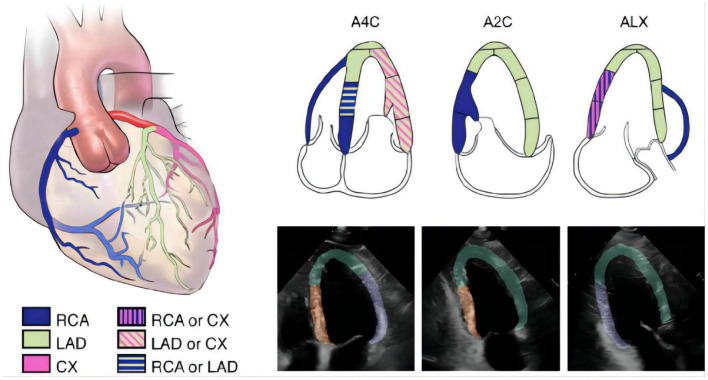
The segmentation of different wall regions. The 2015 ASE guideline recommend typical distributions of the coronary artery in apical four-chamber (A4C), apical two-chamber (A2C), and apical long-axis (ALX) views. In the echocardiographic images, we labeled A for apical, anterior and anteroseptal walls (green area), F for inferior and inferoseptal walls (orange area), and L for anterolateral and inferolateral walls (purple area).

The segmentation model was developed to outline the endocardial and epicardial borders of the left ventricle and the endocardial borders of the left atrium, right atrium and right ventricle. Left ventricle was grouped into 3 different regions, designated A (apical, anterior, and anteroseptal walls), F (inferior and inferoseptal walls) and L (anterolateral and inferolateral walls) according to 2015 American Society of Echocardiography guidelines (19) (Figure 2). We annotated 493 apical 4-chamber videos (8,555 frames), 332 apical 2-chamber videos (5,768 frames) and 366 apical long-axis videos (6,389 frames) which served as ground truth for developing and testing this algorithm to segmented the heart into regions A, F and L as detailed above (19, 20). Myocardial segmentation masks were generated for every frame of each video with the pretrained segmentation LSTM-Unet (21–23). Three separate segmentation models with the same structure were developed to analyze the A4C, A2C, and ALX views. For the A4C video, the model segmented the left ventricle, the left atrium, the right ventricle and the right atrium which were used to quantify the size of each chamber. For detection of RWMAs, each video frame was cropped into a 128 × 128 pixels square with the left ventricle at the center and pixel values are normalized to the range from 0 to 1 (Figure 2).

### Detection of regional wall motion abnormalities

**FIGURE 3:**
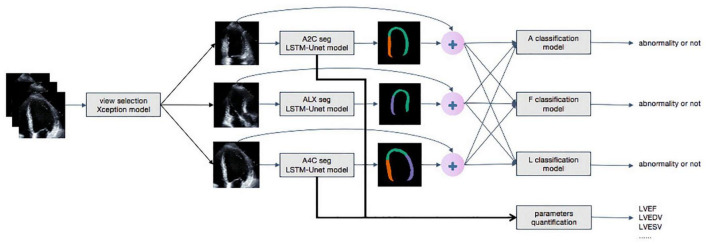
The whole work flow of deep learning model. Steps of data processing. The first model achieves view selection on echocardiography. The Xception model generates a confidence level for view selection and selects A4C, A2C, and ALX views whose confidence is higher than 0.9. Secondly, LSTM-Unet segments each frames of outputs of Xception. The segment and the original video are concatenated as inputs of classification models to detect regional wall motion abnormality. The outputs of LSTM-Unet with A4C and A2C are calculated important parameters, such as LVEDV, LVESV, and LVEF.

The overall process for detecting RWMAs was summarized in Figure 3. Each original DICOM video was concatenated with the mask of the myocardium obtained by the segmentation model. The mask and video were then input into A, F, and L classification models. Detection of the presence of RWMAs and the territory of RWMAs was achieved with Deep 3D Convolution Neural Network.

Details of the RWMA detection model are shown in Supplementary Figure 2. The backbone of the model is R2plus1D, which is a time-saved and calculation-saved feature extractor. In order to effectively use the information extracted by the R2plus1D feature extractor, three fully connected layers are added to the model (24). There is a Batch Normalization layer, an activation layer (LeakyReLU) and a 50% dropout layer following each full-connected layer. Batch Normalization can improve the efficiency of model training, which can save time required to train the video model (25). The output of the RWMA detection model contains two values (two red neurons as shown in Supplementary Figure 2). These values (the score of no abnormality and the score of an abnormality) are derived from the full-connected layers transform information extracted by R2plus1D.

The RWMAs detection models were trained using two Graphics Processing Units (GPU), NVIDIA Tesla P100. Each model contains about 1 million parameters. All the parameters are trained in the direction of minimizing cross entropy, which is an error function to calculate how far the models’ outputs is from real label. The models are trained with Stochastic Gradient Descent Momentum (SGDM) with 0.9 momentum and 1e−4 weight decay. The learning rate starts from 1e−5 and increases linearly with epoch until 1e−4 at epoch 10, which is called warm-up (26). Then learning rate decline linearly from 1e−4 at epoch 10 to 5e−5 at epoch 50.

In order to improve the generalization of RWMAs detection models, spatiotemporal video augmentation methods are adopted. The left subplot in Supplementary Figure 3 shows the clipping in the time dimension and the right subplot shows the spatial cropping. At the training state, each video is randomly cropped and clipped in the spatial and temporal dimensions, so as to enhance the diversity of data and the generalization of the model. In the test and validation phase, the videos are divided into non-overlapping 8-frame video segments and each video segment is inferred three times with three spatial crops. For example, a 32-frame video is divided into 4 video segments, each of which contain 8 frames, and each video segment generates three crops, which means the 32-frame sample generate 12 results in total. Majority voting combines 12 results. The models are performed with Python 3.6.8 and PyTorch 1.4.0. The code will be released in GitHub.

### Quantification of key metrics

The key metrics derived from the model include left ventricle ejection fraction (LV EF), end-diastolic volume (LV EDV), end-systolic volume (LV ESV), end-diastolic transversal dimension (LV EDTD), left atrial end-systolic transversal dimension (LA ESTD), right ventricular end-diastolic transversal dimension (RV EDTD), and right atrial end-systolic transversal dimension (RA ESTD). We calculated these metrics based on the output of segmentation model and the 2015 guidelines of the American Society of Echocardiography and the European Association of Echocardiography (19). In order to enhance the interpretability of deep learning, we adopted the segmentation model to segment the area of four chambers, and then used Simpson biplane method to calculate LVEDV, LVESV, and EF. The long short-term memory (LSTM) can effectively extract the time information from the video.

### Statistical analysis

Analyses were performed using algorithms written in Python 3.6 from the libraries of Numpy, Pandas, and Scikit-learn. Continuous variables were expressed as mean ± standard deviation, median and interquartile range, or counts and percentage, as appropriate. Comparisons of reports and machine algorithm performances were performed using one-way analysis of variance (ANOVA), followed by the least significant difference (LSD) *t*-test. The detection models were assessed according to the area under the receiver operating characteristic (AUROC) curves which plotted sensitivity versus 1–specificity derived from the model’s prediction confidence score. Results were regarded as statistically significant when *P* < 0.05. All calculations were performed by using IBM SPSS version 23.0.

## Result

### Study population

For the internal training and validation dataset, a total of 2,274 transthoracic echocardiographic examinations were divided between standard and bedside ultrasound. As specified in the echocardiography and clinical reports, MIs and RWMAs were present in 1,137 of the 2,274 studies (50%), 62% of which were from bedside ultrasound. In the internal test dataset, MIs and RWMAs were present in 374 of 1,868 cases (20%), 52% of which were examined by bedside ultrasound. In the external test dataset, MIs and RWMAs were present in 849 of 3,026 cases (28%), 37% of which were examined by bedside ultrasound. The clinical and echocardiographic characteristics of the included populations are summarized in Table 1 (training dataset) and Supplementary Table 1 (internal and external test datasets). As expected, significant differences in baseline characteristics existed between normal subjects and those with a myocardial infarction.

**TABLE 1:** Baseline characteristics of the training and validation dataset.

	Training and validation dataset			

	Standard		Bedside	
		
	MI	Normal	MI	Normal
Echo number	430	947	707	190
Age	65 (55,73)	60 (53,76)	67 (54,77)	58 (50,66)
Male patients(%)	353 (83.3)	590 (62.3)	460 (65.2)	118 (62.1)
**Comorbidities (%)**				
Hypertension	115 (35.1)	249 (26.3)	270 (42.4)	37 (19.5)
Hyperlipidemia	217 (66.2)	148 (14.6)	326 (51.3)	10 (5.3)
Diabetes	124 (38.0)	103 (10.9)	324 (50.8)	21 (11.1)
Renal insufficiency	65 (17.4)	79 (8.3)	228 (35.8)	6 (3.2)
Ischemic stroke history	53 (17.4)	96 (10.1)	121 (21.5)	17 (8.9)
**Echo parameters**				
LV EF (%)	46 (41,54) *	62 (60,64)	43 (36,48) †	60 (59,62)
LV EDV (mm^2^)	119 (98,144) *	87 (80,100)	106 (88,129) †	84 (75,98)
LV ESV (mm^2^)	62 (48,82) *	33 (30,37)	59 (46, 76) †	33 (30,39)
LV EDTD (mm)	49 (45,53) *	43 (41,45)	47 (43,51) †	42 (40,45)
LA ESTD (mm)	40 (38,43) *	36 (34,38)	41 (38,43) †	36 (34,38)
RV EDTD (mm)	32 (30,34) *	30 (29,32)	31 (29,33) †	30 (28,32)
RA ESTD (mm)	32 (30,34) *	30 (28,32)	32 (29,34) †	30 (28,31)
**Territories of RWMAs**				
Multiple walls	168 (39.1)		319 (45.1)	
A	291 (67.7)		529 (74.8)	
F	220 (51.2)		363 (51.3)	
L	154 (35.8)		268 (37.9)	

Values are median (IQR) or n (%). *p < 0.05 vs. normal subjects in standard group. †p < 0.05 vs. normal subjects in bedside group. BMI, Body Mass Index; LVEF, left ventricular ejection fraction; LVEDV, left ventricular end-diastolic volume; LVESV, left ventricular end-systolic volume; LV EDTD, left ventricular end-diastolic transversal dimension; LA ESTD, left atrial end-systolic transversal dimension; RV EDTD, right ventricular end-diastolic transversal dimension; RA ESTD, right atrial end-systolic transversal dimension; MI, myocardial infarction; RWMAs, regional wall motion abnormalities; A, apical, anterior and anteroseptal walls; F, inferior and inferoseptal walls; L, anterolateral and inferolateral walls.

### View selection and segmentation

As summarized in Supplementary Figure 4, the deep-learning architecture identified the apical 4-chamber, 2-chamber and long-axis views with a high degree of accuracy: 94, 99, and 95%, respectively. The quality control model achieved an average 95% consistency compared with expert in identifying qualified images (Supplementary Figure 1). As for segmentation, the model provided good agreement with manual segmentation with an average Dice of 0.89 (Table 2). Although the performance of the model for segmenting bedside ultrasound images was slightly lower than that in standard ultrasound, our model was applicable with both machines.

**TABLE 2:** Performance of the segmentation model.

	Segmentation (Dice)									

	LV endocardium		LV myocardium		LA endo		RV endo		RA endo	
					
	Standard	Bedside	Standard	Bedside	Standard	Bedside	Standard	Bedside	Standard	Bedside
A4C	0.94	0.95	0.84	0.81	0.94	0.93	0.89	0.90	0.94	0.93
A2C	0.93	0.93	0.79	0.77	0.93	0.91				
ALX	0.93	0.92	0.82	0.78	0.93	0.93				

A4C, apical 4-chamber; A2C, apical 2-chamber; ALX, apical long axis; LV, left ventricle; LA, left atrium; RA, right atrium; RV, right ventricle; A4C, apical 4-chamber; A2C, apical 2-chamber; endo, endocardium.

### Detection of regional wall motion abnormalities

**FIGURE 4:**
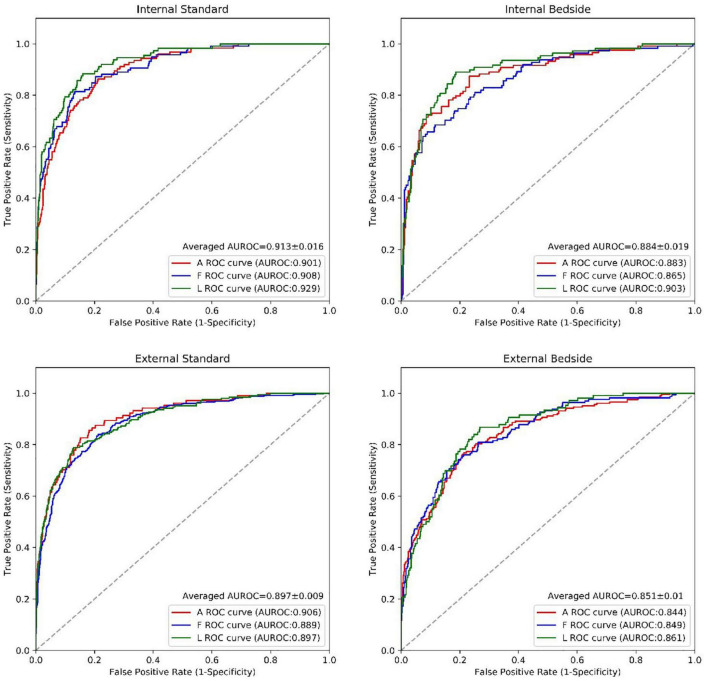
The performance of the RWMAs detection model. The performance of the RWMAs detection model for bedside vs. standard cases in retrospective invalidation dataset and prospective testing dataset. Abbreviations as in Figure 2.

For the detection of a regional wall motion abnormalities in the internal test dataset, the deep learning model had an average AUROC of 0.91 for images obtained with standard echocardiographic equipment compared to 0.88 for images obtained with beside equipment. Youden’s Index was used to evaluate model performance, which yielded sensitivities of 85.4% vs. 85.2% and specificities of 83.2% vs. 78.2% for standard versus beside equipment, respectively. In the external test dataset, the model achieved an average AUROC of 0.90 vs. 0.85 for standard versus bedside ultrasound, with corresponding sensitivities of 81.6% vs. 78.3% and specificities of 83.7% vs. 78.1%. The model had a similar performance for detecting anterior, inferior and lateral wall motion abnormalities in both bedside and standard ultrasound (Figure 4 and Table 3). Overall, these results corresponded to comparable accuracies in detecting RWMAs in the three territories: 0.83 for anterior, 0.81 for inferior and 0.85 for lateral walls.

**TABLE 3:** Performance of model for identifying the presence and territories of RWMAs.

	Internal test dataset		External test dataset	
		
	Standard	Bedside	Standard	Bedside
**AUC**				
A	0.901	0.883	0.906	0.844
F	0.908	0.865	0.889	0.849
L	0.929	0.903	0.897	0.861
Average	0.913	0.884	0.897	0.851
**Sensitivity**				
A	86.3%	87.4%	82.7%	76.80%
F	81.4%	79.3%	83.3%	76.10%
L	88.4%	89.0%	78.9%	82.10%
Average	85.4%	85.2%	81.6%	78.30%
**Specificity**				
A	78.8%	76.8%	84.9%	78.7%
F	86.7%	76.4%	79.3%	78.8%
L	84.0%	81.3%	87.0%	76.9%
Average	83.2%	78.2%	83.7%	78.1%

A, apical, anterior and anteroseptal walls; F, inferior and inferoseptal walls; L, anterolateral and inferolateral walls.

To test the advantages of this tool for experts and beginners, we randomly selected 100 cases from both MI and control cases captured from standard and bedside equipment. In total, 3 experts and 5 beginners participated in the test, where the first reads were based on their own judgments, while they had access to the AI results for the second reads. The second reads were performed at a separate time without access to the results of the first read. The comparison of results of the first and second reads are summarized in Supplementary Figure 5 and Supplementary Table 2. The AI models did not significantly improve the accuracy of experts, but was very helpful for beginners, with average accuracy improving by 9.8, 7.4, and 12.8% for the A, F and L territories, respectively (Supplementary Table 2).

### Quantification of metrics of chamber sizes and function

**FIGURE 5:**
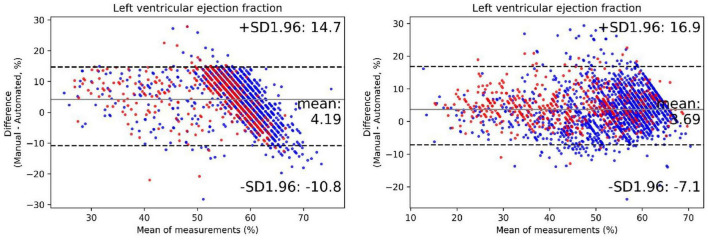
The performance of the automated quantification model. Bland–Altman plots of left ventricular ejection fraction in repeated measurements using the exact same video clips of internal **(left plot)** and external **(right plot)** testing dataset. The red dots represent cases acquired from portable bedside ultrasound; the blue dots represent cases acquired from standard ultrasound. The black lines represent limits of agreement.

The output of our segmentation model was used to compute chamber dimensions and ejection fraction based on the biplane method of disks summation (modified Simpson’s rule) (19). As above, this analysis was performed on studies which passed the automated quality control algorithm. Results of the Bland–Altman analysis comparing parameter values provided by the AI algorithm and from the clinical reports are summarized in Table 4. For each of the 7 parameters, the mean bias and LOAs were similar for the analysis performed on studies obtained with standard and bedside equipment. Accordingly, data are further summarized in the Bland–Altman plots in Figure 5 (LVEF) and Supplementary Figure 6 (structural parameters) in which results obtained from standard and bedside equipment are pooled.

**TABLE 4a:** The measurements of the corresponding clinical metrics for the RWMAs made by physicians and predicted by AI in internal test dataset.

Parameters	Equipment	Median value from clinical report (IQR)	Bland–Altman analysis (Physicians vs. AI)		
			
			Bias	Upper LOA	Lower LOA
LV EF	Standard	60 (59,62)	4.0	15	−11
	Bedside	58 (51,60)	4.7	15	−9
LV EDV	Standard	92 (81,108)	6.0	50	−40
	Bedside	85 (77,101)	6.4	45	−39
LV ESV	Standard	36 (31,43)	−1.1	19	−23
	Bedside	35 (31,47)	−1.2	21	−30
LV EDTD	Standard	44 (42,47)	0.8	8.0	−5.9
	Bedside	42 (38,46)	1.5	11	−6.2
LA ESTD	Standard	38 (35,40)	2.6	14	−7.5
	Bedside	36 (31,41)	2.7	15	−8.0
RV EDTD	Standard	31 (29,33)	−0.9	8.1	−9.5
	Bedside	31 (29,33)	0.9	10	−8.4
RA ESTD	Standard	31 (29,33)	0.5	11	−9.0
	Bedside	32 (29,33)	1.5	11	−10

**TABLE 4b:** The measurements of the corresponding clinical metrics for the RWMAs made by physicians and predicted by AI in external test dataset.

Parameters	Equipment	Median value from clinical report (IQR)	Bland–Altman analysis (Physicians vs. AI)		
			
			Bias	Upper LOA	Lower LOA
LV EF	Standard	59 (51,63)	3.4	17	−7.7
	Bedside	47 (37,58)	4.6	16	−4.1
LV EDV	Standard	103 (95,114)	14	41	−20
	Bedside	108 (90,136)	4.7	58	−43
LV ESV	Standard	59 (55,62)	6.6	16	−12
	Bedside	55 (39,83)	−2.2	18	−24
LV EDTD	Standard	48 (45,50)	1.9	12	−6.4
	Bedside	48 (43,53)	−1.1	7.0	−10
LA ESTD	Standard	36 (32,39)	−1.4	10	−12
	Bedside	40 (36,44)	0.5	12	−14
RV EDTD	Standard	35 (32,38)	1.8	11	−7.6
	Bedside	35 (21,38)	1.2	8.9	−7.5
RA ESTD	Standard	35 (32,38)	1.4	9.8	−5.9
	Bedside	35 (31,39)	−0.1	13	−9.7

LVEF, left ventricular ejection fraction; LVEDV, left ventricular end-diastolic volume; LVESV, left ventricular end-systolic volume; LV EDTD, left ventricular end-diastolic transversal dimension; LA ESTD, left atrial end-systolic transversal dimension; RV EDTD, right ventricular end-diastolic transversal dimension; RA ESTD, right atrial end-systolic transversal dimension; IQR, interquartile range; LOA, limits of agreement.

We further extended our analysis to segregate patients into 3, clinically meaningful discrete LVEF groups: reduced (<40%), midrange (40–50%) and preserved (>50%). The results of this prediction were moderately consistent with that of echocardiographic reports, with an accuracy of 77% (Supplementary Table 3). There was a tendency to underestimate LVEF in our model, especially in higher manual values. Overall, these results indicate that the degree of accuracy of the automatic quantification of these key metrics was within the bounds of normal clinical practice.

## Discussion

Prompt recognition of RWMAs by echocardiography is an important tool for timely diagnosis and treatment of myocardial infarction in patients presenting with chest pain, especially in the emergency department. However, accurate diagnosis relies on technical expertise in image acquisition, intrinsic quality of the imaging equipment, and significant experience in image interpretation. Technological advances in portable echocardiographic equipment are making high quality imaging more readily available. However, availability of appropriately trained physicians for on-demand interpretation is limited in most hospitals and analyses performed by less experienced physicians may lead to misdiagnoses which can adversely impact clinical care. Our tool provides a fully automated pipeline for all routine aspects of interpreting echocardiograms. For example, echocardiographic images obtained from a patient admitted to the emergency department with chest pain can be submitted electronically to the model which automatically assess for the presence of RWMAs and also quantifies cardiac function, enabling high-efficient serial primary care. In non-emergent settings, this tool can be used to assess temporal changes of regional and global heart function during repeated echocardiographic videos in patients during follow up for a myocardial infarction, for monitoring cardiotoxicity during chemotherapy and in patients receiving cardiac rehabilitation. Our model makes analysis of these echocardiograms less burdensome to the system while maintaining (or even enhancing) reliability and reproducibility.

Our study is the first to rigorously demonstrate that deep learning methods can automatically assess image quality and interpret RWMAs with a high degree of accuracy and to provide a comparison of results from standard and portable echocardiographic equipment. The first steps in our pipeline involve automated view selection, quality control and image segmentation. Each of these steps was performed with a high degree of accuracy. The importance of automated image quality assessment cannot be overstated. In order to mimic clinical practice, we did not apply any initial screening of image quality for inclusion since physicians are also faced with images of varied quality. Our algorithm excluded unqualified images from which detection of RWMAs would be inappropriate, even by experienced clinicians (see Supplementary Figure 1 for examples). Interestingly, 2.7% of standard cases and 14.7% of portable bedside cases were excluded. As summarized Supplementary Figure 7, when the deep learning model was applied to detect regional wall motion abnormalities in these unqualified images, the AUCs, sensitivities and specificities were all markedly decreased and the bias and LOAs for each of the 7 parameters of chamber sizes and function were significantly larger. In our cohort, most portable bedside studies were obtained in the emergency room in patients presenting with chest pain. Thus, the higher rate of exclusion of bedside cases may reflect factors such as the critical nature of the patients under which images are obtained and perhaps less availability of experienced sonographers in this setting. Under such urgent conditions, less consideration may be given to image quality. Therefore, availability of an AI model that can provide feedback in real time can promote acquisition of high-quality images and ensure that measurements and detection of RWMAs are based on qualified images.

Another important feature of our model that analyzed RWMAs was that it focused analysis on the left ventricle by excluding the other cardiac chambers. As such, the segmentation model (which achieved an average Intersection Over Union value of 80.9%) was able to divide the left ventricle into three regions corresponding to coronary artery perfusion territories (15, 16). This division was based on the current guidelines and, in addition to its intrinsic clinical utility, could provide the foundation for subsequent research. The model exhibited a high performance with average Intersection Over Union value of 80.9%, but relatively lower for epicardium due to the obscure borders near the edge of imaging area.

### Wall motion abnormality detection and classification

Overall, the deep learning model exhibited good performance with similar accuracies for detecting RWMAs in all 3 regions of the left ventricle in both internal and external test datasets (Table 3). Performance in the external test dataset was only slightly lower (by ∼3%) than in the external test dataset. Also importantly, results achieved from images obtained with the bedside ultrasound were comparable to those of the standard equipment with the difference of average AUC between equipment of only 0.04 in internal and external test datasets (Figure 4). Our primary motivation is that the model will assist, not replace, physician decision making. Therefore, the AI model will save experts’ time without influence his or her judgment and proved to be very helpful for beginners, with average accuracy improving by 9.8, 7.4, and 12.8% respectively for the A, F, and L territories. With the advantage of objectivity and consistency, the AI model may become an educational tool for beginners to improve their skill in image acquisition and interpretation.

We also analyzed the incorrect cases of each models using Logistic regression. After multivariable adjustment, correlation between the accuracy and age was statistically significant in all models (Supplementary Table 4). The violin plot showed that the average age of incorrect cases was older than that of correct cases (Supplementary Figure 8). This finding is consistent with our experience in clinical practice. Because the degree of wall motion in older patients was generally lower than young patients, which makes it more difficult for models to distinguish MI and normal cases.

### Automatic quantification of cardiac function

In addition to detection of territories with RWMAs, patient care is influenced by parameters of cardiac size and function. Accordingly, our model also automatically and reliably quantified the relevant parameters derived from end-diastolic and end-systolic images. Since it is intended that our deep learning model be used in conjunction with bedside echocardiographic devices without ECG capabilities, end-diastolic and end-systolic images need to be selected based on endocardial areas determined from the segmentation model; this approach is similar to those employed by Zhang et al. (10) and Ouyang et al. (9). Finally, the deep learning model was reasonably consistent with physicians’ classification of reduced (<40%), midrange (40∼50%) and preserved (>50%) LVEFs, which has important implications for treatment and prognosis of patients with heart failure (3, 5).

### Related work

Automated detection of RWMAs have been described in two recent studies. Kusunose et al constructed a deep learning model that utilized 3 mid-level short-axis static images acquired at the end-diastolic, mid-systolic, and end-systolic phases to detect the presence and territories of RWMAs (15). The highest AUC produced by the model is 0.97, which is similar to the AUC by cardiologist and significantly higher than the AUC by resident readers. However, the pipeline was semiautomatic in that the initial input requires manual selection for echocardiogram views and cycle phases. As such, that model was based on analysis of static images, which does not parallel how RWMAs are detected in clinical practice which rely on dynamic videos. Finally, the study lacked external test dataset.

Huang et al also developed a deep learning model for detection of RWMA that directly analyzed dynamic videos, first by performing automated view selection and segmentation (16). The AUC for the external dataset was 0.89. However, the dataset of RWMAs was relatively small (*n* = 576) and 84% (*n* = 486) of studies included RWMAs of multiple walls; thus the ability to detect cases with single wall RWMAs was not fully evaluated. In contracts, nearly 50% of cases in our study have single wall RWAMs. In addition, to meet the stringent quality control, the author excluded up to a third of the examinations despite only using standard echocardiographic equipment. This model is therefore not suitable for widespread use in clinical practice, especially for analysis of bedside ultrasound in the emergency department. In contrast, our quality control algorithm was based on assessments by expert echocardiographers and therefore more closely mimicked clinical practice. Accordingly, our algorithm excluded only 2.7% of studies from standard equipment and 14.7% of studies from portable bedside studies. Despite having excluded a smaller percent of cases, our overall model performed was comparable to that of this prior study. Thus, our fully automatic pipeline can be applied to both standard and bedside ultrasound for detection of RWMAs and measurement of cardiac function, even in the emergency department.

## Study limitations

The results of our study need to be considered within the context of several limitations. First, the distribution of echocardiography machines differed between MI and control cases, because the MI cases are more likely to have been performed with portable bedside equipment in an intensive care or emergency department, while control cases were mainly obtained by standard equipment in dedicated ultrasound rooms. Second, like other deep learning studies, we face the “black box problem” related to unexplained model features and how they contribute to the final result. To limit this problem to some degree, we removed irrelevant areas (e.g., RV free wall region) during the segmentation process so that the model focused on LV myocardium. Third, our model detected the presence and RWMAs rather than the severity of motion abnormalities, because wall motion score indexes recommended by society guidelines have interobserver and intra-observer variabilities. Instead, we developed an automated model to quantify cardiac function in real time. Although our model achieved good performance in the external test set, testing of the pipeline in a prospective RCT cohort is warranted.

## Conclusion

We developed and validated a fully automated echocardiography pipeline applicable to both standard and portable bedside ultrasound with various functions, including view selection, quality control, segmentation, detection of the region of wall motion abnormalities and quantification of cardiac function. With high levels of sensitivity and specificity, the model has the potential to be used as a screening tool to aid physician in identifying patients with RWMAs, particularly in through the use of portable bedside ultrasound in the emergency room and intensive care units.

## Data availability statement

The original contributions presented in this study are included in the article/Supplementary material, further inquiries can be directed to the corresponding author.

## Author contributions

KH, XXL, and FY designed this study, analyzed and interpreted the patient data, drafted the manuscript, and guaranteed that all aspects of the work was investigated and resolved. DB and KH critically revised the important intellectual content of the manuscript. WW, XuC, QW, LZ, HG, BL, LY, and XL collected and analyzed and interpreted the data. YC, XiC, HP, PZ, and ZW designed the network architecture and performed the data preparation and analysis. All authors read and approved the final manuscript.

## Abbreviations

A4C: apical four chambers
A2C: apical two chambers
ALX: apical long axis
CNN: convolutional neural networks
DL: deep learning
LVEF: left ventricular ejection fraction
LVEDV: left ventricular end-diastolic volume
LVESV: left ventricular end-systolic volume
LV EDTD: left ventricular end-diastolic transversal dimension
LA ESTD: left atrial end-systolic transversal dimension
LOA: limits of agreement
MI: myocardial infarction
RV EDTD: right ventricular end-diastolic transversal dimension
RA ESTD: right atrial end-systolic transversal dimension
RWMAs: regional wall motion abnormalities
ROC: receiver operating characteristic.

## References

[1] Shengshou H. Report on cardiovascular health and diseases in China 2021: an updated summary. *Chin Circ J.* (2022) 37:26. 10.3967/bes2024.162 39401992

[2] Xu H Yang Y Wang C Yang J Li W Zhang X Association of hospital-level differences in care with outcomes among patients with acute st-segment elevation myocardial infarction in China. *JAMA Netw Open.* (2020) 3:e2021677. 10.1001/jamanetworkopen.2020.21677 33095249 PMC7584928

[3] Vogel B Claessen BE Arnold SV Chan D Cohen DJ Giannitsis E St-segment elevation myocardial infarction. *Nat Rev Dis Primers.* (2019) 5:39. 10.1038/s41572-019-0090-3 31171787

[4] Prastaro M Pirozzi E Gaibazzi N Paolillo S Santoro C Savarese G Expert review on the prognostic role of echocardiography after acute myocardial infarction. *J Am Soc Echocardiogr.* (2017) 30:431–43.e2. 10.1016/j.echo.2017.01.020 28477781

[5] Prasad SB Lin AK Guppy-Coles KB Stanton T Krishnasamy R Whalley GA Diastolic dysfunction assessed using contemporary guidelines and prognosis following myocardial infarction. *J Am Soc Echocardiogr.* (2018) 31:1127–36. 10.1016/j.echo.2018.05.016 30097298

[6] Amsterdam EA Wenger NK Brindis RG Casey DE Jr. Ganiats TG Holmes DR Jr. 2014 Aha/Acc guideline for the management of patients with non-st-elevation acute coronary syndromes: a report of the American college of cardiology/American heart association task force on practice guidelines. *J Am Coll Cardiol.* (2014) 64:e139–228. 10.1016/j.jacc.2014.09.017 25260718

[7] Ibanez B James S Agewall S Antunes MJ Bucciarelli-Ducci C Bueno H 2017 esc guidelines for the management of acute myocardial infarction in patients presenting with st-segment elevation: the task force for the management of acute myocardial infarction in patients presenting with st-segment elevation of the European Society of Cardiology (Esc). *Eur Heart J.* (2018) 39:119–77. 10.1093/eurheartj/ehx393 28886621

[8] Parisi AF Moynihan PF Folland ED Feldman CL. Quantitative detection of regional left ventricular contraction abnormalities by two-dimensional echocardiography. Ii. Accuracy in coronary artery disease. *Circulation.* (1981) 63:761–7. 10.1161/01.cir.63.4.761 7471330

[9] Ouyang D He B Ghorbani A Yuan N Ebinger J Langlotz CP Video-based ai for beat-to-beat assessment of cardiac function. *Nature.* (2020) 580:252–6. 10.1038/s41586-020-2145-8 32269341 PMC8979576

[10] Zhang J Gajjala S Agrawal P Tison GH Hallock LA Beussink-Nelson L Fully automated echocardiogram interpretation in clinical practice. *Circulation.* (2018) 138:1623–35. 10.1161/CIRCULATIONAHA.118.034338 30354459 PMC6200386

[11] Narula S Shameer K Salem Omar AM Dudley JT Sengupta PP. Machine-learning algorithms to automate morphological and functional assessments in 2d echocardiography. *J Am Coll Cardiol.* (2016) 68:2287–95. 10.1016/j.jacc.2016.08.062 27884247

[12] Kusunose K Haga A Inoue M Fukuda D Yamada H Sata M. Clinically feasible and accurate view classification of echocardiographic images using deep learning. *Biomolecules.* (2020) 10:665. 10.3390/biom10050665 32344829 PMC7277840

[13] Silva JF Silva JM Guerra A Matos S Guerra A Costa C. Ejection fraction classification in transthoracic echocardiography using a deep learning approach. *Proceedings of the 2018 IEEE 31st International Symposium on Computer-Based Medical Systems (CBMS).* Karlstad: (2018).

[14] Yue Z Li W Jing J Yu J Yi S Yan W. Automatic Segmentation of the Epicardium and Endocardium Using Convolutional Neural Network. *Proceedings of theIEEE International Conference on Signal Processing.* Chengdu: IEEE (2017).

[15] Kusunose K Abe T Haga A Fukuda D Yamada H Harada M A deep learning approach for assessment of regional wall motion abnormality from echocardiographic images. *JACC Cardiovasc Imaging.* (2020) 13(2 Pt 1):374–81. 10.1016/j.jcmg.2019.02.024 31103590

[16] Huang MS Wang CS Chiang JH Liu PY Tsai WC. Automated recognition of regional wall motion abnormalities through deep neural network interpretation of transthoracic echocardiography. *Circulation.* (2020) 142:1510–20. 10.1161/CIRCULATIONAHA.120.047530 32964749

[17] Sengupta PP Shrestha S Berthon B Messas E Donal E Tison GH Proposed requirements for cardiovascular imaging-related machine learning evaluation (Prime): a checklist: reviewed by the American college of cardiology healthcare innovation council. *JACC Cardiovasc Imaging.* (2020) 13:2017–35. 10.1016/j.jcmg.2020.07.015 32912474 PMC7953597

[18] Yang F Chen X Lin X Chen X Wang W Liu B Automated analysis of doppler echocardiographic videos as a screening tool for valvular heart diseases. *JACC Cardiovasc Imaging.* (2021) 15:551–63. 10.1016/j.jcmg.2021.08.015 34801459

[19] Lang RM Badano LP Mor-Avi V Afilalo J Armstrong A Ernande L Recommendations for cardiac chamber quantification by echocardiography in adults: an update from the American society of echocardiography and the European association of cardiovascular imaging. *J Am Soc Echocardiogr.* (2015) 28:1–39.e14. 10.1016/j.echo.2014.10.003 25559473

[20] Voigt JU Pedrizzetti G Lysyansky P Marwick TH Houle H Baumann R Definitions for a common standard for 2d speckle tracking echocardiography: consensus document of the eacvi/ase/industry task force to standardize deformation imaging. *Eur Heart J Cardiovasc Imaging.* (2015) 16:1–11. 10.1093/ehjci/jeu184 25525063

[21] Falk T Mai D Bensch R Çiçek Ö Abdulkadir A Marrakchi Y U-Net: deep learning for cell counting, detection, and morphometry. *Nat Methods.* (2019) 16:67–70. 10.1038/s41592-018-0261-2 30559429

[22] Ibtehaz N Rahman MS. Multiresunet : rethinking the U-Net architecture for multimodal biomedical image segmentation. *Neural Netw.* (2020) 121:74–87. 10.1016/j.neunet.2019.08.025 31536901

[23] Ronneberger O Fischer P Brox T. *U-Net: Convolutional Networks for Biomedical Image Segmentation.* Cham: Springer (2015).

[24] Du T Wang H Torresani L Ray J Lecun Y Paluri M. A closer look at spatiotemporal convolutions for action recognition. *Proceedings of the IEEE/CVF Conference on Computer Vision and Pattern Recognition.* Salt Lake City, UT: IEEE (2018).

[25] Ioffe S Szegedy C. Batch normalization: accelerating deep network training by reducing internal covariate shift. *Proceedings of the 32nd International Conference on Machine Learning.* Lille: (2015). 10.1007/s11390-020-0679-8

[26] Goyal P Dollár P Girshick R Noordhuis P Wesolowski L Kyrola A Accurate, large minibatch sgd: training imagenet in 1 hour. *arXiv[Preprint].* (2017). Available online at: 10.48550/arXiv.1706.02677 (accessed June 8, 2017).35895330

