# Supplementary material for: Echocardiography-based AI detection of regional wall motion abnormalities and quantification of cardiac function in myocardial infarction
Source: Front Cardiovasc Med. 2022 Aug 22;9:903660. doi: 10.3389/fcvm.2022.903660 (PMC9441592; doi:10.3389/fcvm.2022.903660)

**Supplemental Material**

**Table of Contents**

**Table S1………………………………………………………………………………2**

**Table S2……………………………………………………………………………… 3**

**Table S3………………………………………………………………………………5**

**Table S4……………………………………………………………………………… 6**

**Supplemental figure legends……………………………………………………….10**

**Figure S1…………………………………………………………………………….11**

**Figure S2…………………………………………………………………………….12**

**Figure S3…………………………………………………………………………….13**

**Figure S4…………………………………………………………………………….14**

**Figure S5…………………………………………………………………………….15**

**Figure S6…………………………………………………………………………….16**

**Figure S7…………………………………………………………………………….17**

**Figure S8…………………………………………………………………………….18**

**Table S1a.** Baseline Characteristics of the Internal Test Dataset

|  | **Internal Test Dataset** | | | |
| --- | --- | --- | --- | --- |
|  | **Standard** | | **Bedside** | |
|  | **MI** | **Normal** | **MI** | **Normal** |
| Echo number | 195 | 1227 | 179 | 267 |
| Age | 65 (56,73) | 58 (47,68) | 71 (59,83) | 71 (58,84) |
| Male patients（%） | 174 (76.3) | 728 (59.3) | 203 (74.1) | 225 (55.3) |
| Comorbidities (%) |  |  |  |  |
| Hypertension | 78 (40.0) | 206 (16.8) | 80 (44.7) | 61 (14.6) |
| Hyperlipidemia | 116 (59.5) | 79 (6.4) | 101 (56.4) | 26 (6.2) |
| Diabetes | 95 (48.7) | 78 (6.4) | 98 (54.7) | 41 (9.8) |
| Renal insufficiency | 43 (22.1) | 66 (5.4) | 54 (30.2) | 16 (3.8) |
| Ischemic stroke history | 36 (18.5) | 119 (9.7) | 30 (16.8) | 24 (9.0) |
| Echo parameters |  |  |  |  |
| LV EF (%) | 48 (41,56) ***** | 61 (60,63) | 43 (36,48) **†** | 59 (58,60) |
| LV EDV (mm^2^) | 112 (96,134) ***** | 90 (80,103) | 88 (60,108) **†** | 80 (70,89) |
| LV ESV (mm^2^) | 57 (43,76) ***** | 35 (31,40) | 48 (38,65) **†** | 33 (30,36) |
| LV EDTD (mm) | 49 (45,53) ***** | 43 (42,46) | 48 (45,53) **†** | 45 (41,48) |
| LA ESTD (mm) | 40 (38,43) ***** | 37 (35,39) | 40 (37,46) **†** | 38 (36,42) |
| RV EDTD (mm) | 32 (30,34) ***** | 30 (29,32) | 31 (30,34) | 31 (28,33) |
| RA ESTD (mm) | 33 (31,35)***** | 31 (29,33) | 33 (30,35) **†** | 31 (29,33) |
| Territories of RWMAs |  |  |  |  |
| Multiple walls | 118 (58.7) |  | 149 (54.0) |  |
| A | 126 (62.7) |  | 189 (68.5) |  |
| F | 123 (61.2) |  | 158 (57.2) |  |
| L | 117 (58.2) |  | 145 (52.5) |  |

**Table S1b.** Baseline Characteristics of the ExternalTest Dataset

|  | **External Test Dataset** | | | |
| --- | --- | --- | --- | --- |
|  | **Standard** | | **Bedside** | |
|  | **MI** | **Normal** | **MI** | **Normal** |
| Echo number | 535 | 1637 | 314 | 325 |
| Age | 62 (55,72) | 54 (45,63) | 67 (55,76) | 61 (51,77) |
| Male patients（%） | 350 (65.4) | 991 (60.5) | 206 (65.6) | 210 (64.6) |
| Comorbidities (%) |  |  |  |  |
| Hypertension | 298 (55.7) | 526 (32.1) | 179 (57.0) | 132 (40.6) |
| Hyperlipidemia | 108 (20.2) | 155 (9.4) | 97 (30.8) | 65 (20.0) |
| Diabetes | 151 (28.2) | 242 (14.8) | 116 (36.9) | 70 (21.5) |
| Renal insufficiency | 106 (19.8) | 167 (10.2) | 56 (17.8) | 39 (12.0) |
| Ischemic stroke history | 109 (20.4) | 156 (9.5) | 38 (12.1) | 35 (10.8) |
| Echo parameters |  |  |  |  |
| LV EF (%) | 46 (40,51) ***** | 61(58,64) | 42 (35,48) **†** | 55 (46,61) |
| LV EDV (mm^2^) | 119 (111,128) ***** | 99 (93,106) | 115 (97,149) **†** | 103 (84,138) |
| LV ESV (mm^2^) | 60 (57,65) ***** | 38 (36,41) | 67 (54,92) **†** | 44 (34,75) |
| LV EDTD (mm) | 49 (46,53) ***** | 47 (44,50) | 49 (45,53) **†** | 48 (44,56) |
| LA ESTD (mm) | 38 (34,41) ***** | 35 (32,38) | 40 (36,44) **†** | 41 (36,46) |
| RV EDTD (mm) | 35 (33,38) ***** | 35 (32,37) | 34 (31,38) **†** | 36 (32,40) |
| RA ESTD (mm) | 36 (33,39) ***** | 35 (32,38) | 34 (30,38) **†** | 36 (32,40) |
| Territories of RWMAs |  |  |  |  |
| Multiple walls | 223 (41.6) |  | 119 (37.9) |  |
| A | 308 (57.6) |  | 203 (64.6) |  |
| F | 241 (45.0) |  | 163 (51.9) |  |
| L | 230 (43.0) |  | 106 (33.8) |  |

Values are median (IQR) or n (%). *p < 0.05 vs. normal subjects in standard group. †p < 0.05 vs. normal subjects in bedside group.BMI, Body Mass Index; LVEF, left ventricular ejection fraction; LVEDV, left ventricular end-diastolic volume; LVESV, left ventricular end-systolic volume; LV EDTD, left ventricular end-diastolic transversal dimension; LA ESTD, left atrial end-systolic transversal dimension; RV EDTD, right ventricular end-diastolic transversal dimension; RA ESTD, right atrial end-systolic transversal dimension; MI, myocardial infarction; RWMAs, regional wall motion abnormalities; A, apical, anterior and anteroseptal walls; F, inferior and inferoseptal walls; L, anterolateral and inferolateral walls.

**Table S2.** The Accuracy of Experts and Beginners in Detecting MI with or without AI.

|  | A  accuracy | F  accuracy | L  accuracy | A  change | F  change | L  change |
| --- | --- | --- | --- | --- | --- | --- |
| AI | 0.83 | 0.81 | 0.85 |  |  |  |
| expert1 | 0.89 | 0.87 | 0.90 |  |  |  |
| expert1 with AI | 0.88 | 0.88 | 0.89 | -0.01 | 0.01 | -0.01 |
| expert2 | 0.91 | 0.84 | 0.82 |  |  |  |
| expert2 with AI | 0.91 | 0.83 | 0.83 | 0 | -0.01 | 0.01 |
| expert3 | 0.90 | 0.79 | 0.85 |  |  |  |
| expert3 with AI | 0.91 | 0.80 | 0.86 | 0.01 | 0.01 | 0.01 |
| beginner 1 | 0.67 | 0.72 | 0.68 |  |  |  |
| beginner 1 with AI | 0.88 | 0.81 | 0.78 | 0.21 | 0.09 | 0.1 |
| beginner 2 | 0.84 | 0.79 | 0.81 |  |  |  |
| beginner 2 with AI | 0.87 | 0.81 | 0.81 | 0.03 | 0.02 | 0 |
| beginner 3 | 0.87 | 0.69 | 0.70 |  |  |  |
| beginner 3 with AI | 0.90 | 0.80 | 0.79 | 0.03 | 0.11 | 0.09 |
| beginner 4 | 0.69 | 0.68 | 0.58 |  |  |  |
| beginner 4 with AI | 0.84 | 0.74 | 0.83 | 0.15 | 0.06 | 0.25 |
| beginner 5 | 0.79 | 0.67 | 0.65 |  |  |  |
| beginner 5 with AI | 0.86 | 0.76 | 0.85 | 0.07 | 0.09 | 0.2 |

**Table S3.** The Confusion Matrix of Model Prediction and Clinical Reports

| **Prediction**  **Reports** | **EF < 40%** | **EF=40~50%** | **EF >50%** |
| --- | --- | --- | --- |
| **EF < 40%** | 67 | 16 | 3 |
| **EF=40~50%** | 60 | 58 | 39 |
| **EF > 50%** | 13 | 304 | 1308 |

EF, left ventricular ejection fraction

**Table S4a.** Beta Coefficients for the Association Between the Performance of RWMAs Detection Model for A Territories and Clinical Variables Through Logistic Regression Analysis

|  | **Univariableanalysis** | | **Multi-variableanalysis** | |
| --- | --- | --- | --- | --- |
|  | **Standardizedbeta**  **(95% CI)** | **p value** | **Standardizedbeta**  **(95% CI)** | **p value** |
| Age | 1.036 (1.024~1.049) | 0.000 | 1.035 (1.023~1.046) | 0.000 |
| Gender | 1.100 (0.764~1.583) | 0.608 |  |  |
| Comorbidities |  | | | |
| Hypertension | 0.923 (0.613~1.391) | 0.702 |  |  |
| Hyperlipidemia | 1.084 (0.627~1.874) | 0.773 |  |  |
| Diabetes | 1.920 (1.158~3.184) | 0.011 | 1.990 (1.214~3.263) | 0.006 |
| Renal insufficiency | 2.217 (0.969~5.074) | 0.059 | 2.351 (1.065~5.190) | 0.034 |
| Ischemic stroke | 0.922 (0.307~2.766) | 0.885 |  |  |
| Echo parameters |  | | | |
| LV EF (%) | 0.987 (0.947~1.029) | 0.547 |  |  |
| LV EDV (mm^2^) | 1.001 (0.994~1.008) | 0.862 |  |  |
| LV ESV (mm^2^) | 0.991 (0.969~1.013) | 0.424 |  |  |
| LV EDTD (mm) | 1.039 (0.974~1.108) | 0.250 |  |  |
| LA ESTD (mm) | 1.001 (0.944~1.061) | 0.985 |  |  |
| RV EDTD (mm) | 1.053 (0.976~1.137) | 0.181 | 1.071 (1.015~1.131) | 0.013 |
| RA ESTD (mm) | 0.995 (0.922~1.073) | 0.895 |  |  |

|  | **Univariableanalysis** | | **Multi-variableanalysis** | |
| --- | --- | --- | --- | --- |
|  | **Standardizedbeta**  **(95% CI)** | **p value** | **Standardizedbeta**  **(95% CI)** | **p value** |
| Age | 1.041 (1.030~1.052) | 0.000 | 1.041 (1.03~1.052) | 0.000 |
| Gender | 0.928 (0.674~1.278) | 0.647 |  |  |
| Comorbidities |  | | | |
| Hypertension | 0.906 (0.630~1.303) | 0.595 |  |  |
| Hyperlipidemia | 0.989 (0.595~1.645) | 0.966 |  |  |
| Diabetes | 1.624 (1.004~2.628) | 0.048 | 1.610 (1.009~2.569) | 0.046 |
| Renal insufficiency | 1.714 (0.774~3.798) | 0.184 |  |  |
| Ischemic stroke | 0.691 (0.248~1.922) | 0.479 |  |  |
| Echo parameters |  | | | |
| LV EF (%) | 1.063 (1.023~1.105) | 0.002 | 1.047 (1.019~1.075) | 0.001 |
| LV EDV (mm^2^) | 1.003 (0.997~1.009) | 0.364 |  |  |
| LV ESV (mm^2^) | 1.007 (0.988~1.025) | 0.488 |  |  |
| LV EDTD (mm) | 1.030 (0.973~1.091) | 0.309 | 1.050 (1.002~1.102) | 0.042 |
| LA ESTD (mm) | 1.062 (1.009~1.118) | 0.022 | 1.073 (1.024~1.124) | 0.003 |
| RV EDTD (mm) | 1.048 (0.980~1.121) | 0.172 | 1.061 (1.004~1.122) | 0.035 |
| RA ESTD (mm) | 1.027 (0.960~1.098) | 0.442 |  |  |

**Table S4b.** Beta Coefficients for the Association Between the Performance of RWMAs Detection Model for F Territories and Clinical Variables Through Logistic Regression Analysis

**Table S4c.** Beta Coefficients for the Association Between the Performance of RWMAs Detection Model for L Territories and Clinical Variables Through Logistic Regression Analysis

|  | **Univariableanalysis** | | **Multi-variableanalysis** | |
| --- | --- | --- | --- | --- |
|  | **Standardizedbeta**  **(95% CI)** | **p value** | **Standardizedbeta**  **(95% CI)** | **p value** |
| Age | 1.028 (1.015~1.042) | 0.000 | 1.031 (1.018~1.043) | 0.000 |
| Gender | 1.468 (0.955~2.256) | 0.080 | 1.517 (1.013~2.272) | 0.043 |
| Comorbidities |  | | | |
| Hypertension | 1.065 (0.684~1.658) | 0.781 |  |  |
| Hyperlipidemia | 1.189 (0.667~2.120) | 0.557 |  |  |
| Diabetes | 1.420 (0.798~2.529) | 0.233 |  |  |
| Renal insufficiency | 1.651 (0.680~4.011) | 0.268 |  |  |
| Ischemic stroke | 0.265 (0.035~2.013) | 0.199 | 0.212 (0.027~1.654) | 0.139 |
| Echo parameters |  | | | |
| LV EF (%) | 0.989 (0.946~1.034) | 0.635 |  |  |
| LV EDV (mm^2^) | 1.001 (0.994~1.008) | 0.723 |  |  |
| LV ESV (mm^2^) | 0.986 (0.964~1.009) | 0.232 |  |  |
| LV EDTD (mm) | 1.107 (1.031~1.189) | 0.005 | 1.089 (1.05~1.128) | 0.000 |
| LA ESTD (mm) | 1.020 (0.956~1.089) | 0.543 |  |  |
| RV EDTD (mm) | 0.985 (0.905~1.071) | 0.721 |  |  |
| RA ESTD (mm) | 1.022 (0.940~1.111) | 0.610 |  |  |

**Supplemental figure legends**

**Figure S1.** The Examples of Qualified and Unqualified Images and The Performance of Image Quality Control Model

A4C, apical four-chamber view; A2C, apical two-chamber view; ALX, apical long-axis view.

**Figure S2.** The Architecture for RWMAs Detection Model

The architecture of RMWA classification models. The model contains a R2plus1D extractor and a full-connected module: the former extracts the information from the video, and the latter integrates the information to output the probability of motion abnormality.

**Figure S3.** Spatiotemporal Video Augmentation

Spatiotemporal video augmentation in reasoning stage. A video is clipped in temporal dimension into 4 parts and cropped in spatial dimension into 3 patches. Majority voting combines 12 results.

**Figure S4.** The Performance of View Classification Model

The deep-learning architecture identified standard apical views (A4C, A2C and ALX) with a high degree of accuracy (respectively 94%, 99% and 95%). Abbreviations as in Figure 1.

**Figure S5.** The Performance of Experts and Beginners in Detecting RWMAs with or without AI.

**Figure S6.**The Performance of the Automated Quantification Model in Internal and External Test Dataset

The left and right plots were respectively the results in internal and external test dataset; the red dots represent cases acquired from portable bedside ultrasound; the blue dots represent cases acquired from standard ultrasound; the black lines represent limits of agreement.

**Figure S7.** The Performance of RWMAs Detection Model and AutomaticQuantification Model in the Unqualified Dataset

The red dots represent cases acquired from portable bedside ultrasound; the blue dots represent cases acquired from standard ultrasound. The black lines represent limits of agreement. Abbreviations as in Figure 2.

**Figure S8.** The distribution of age in correct and incorrect cases.

**Figure S1.**The Examples of Qualified and Unqualified Images and The Performance of Image Quality Control Model


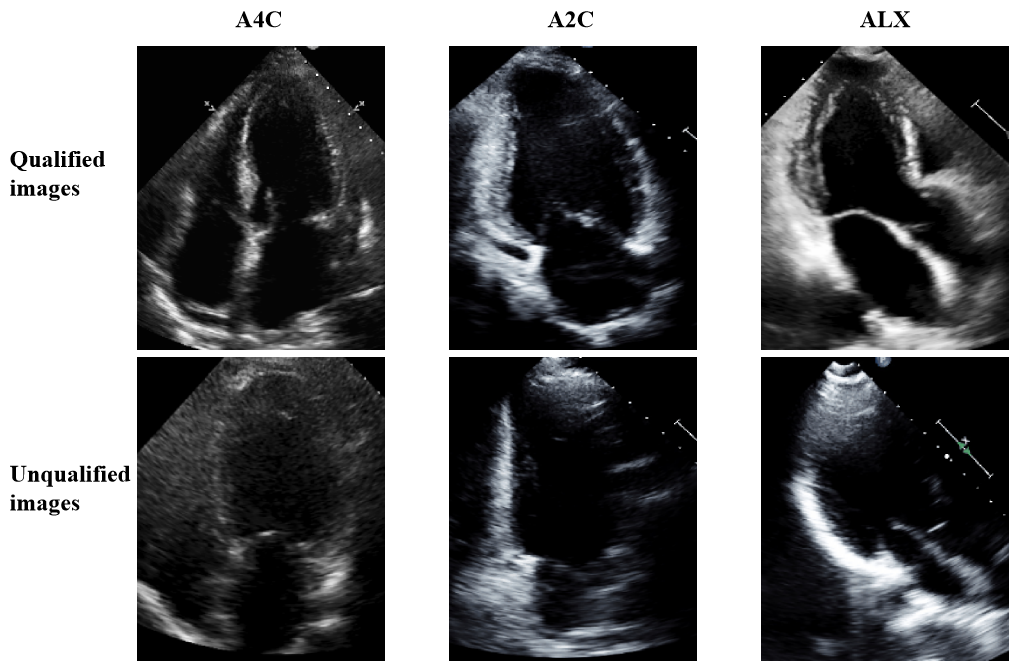

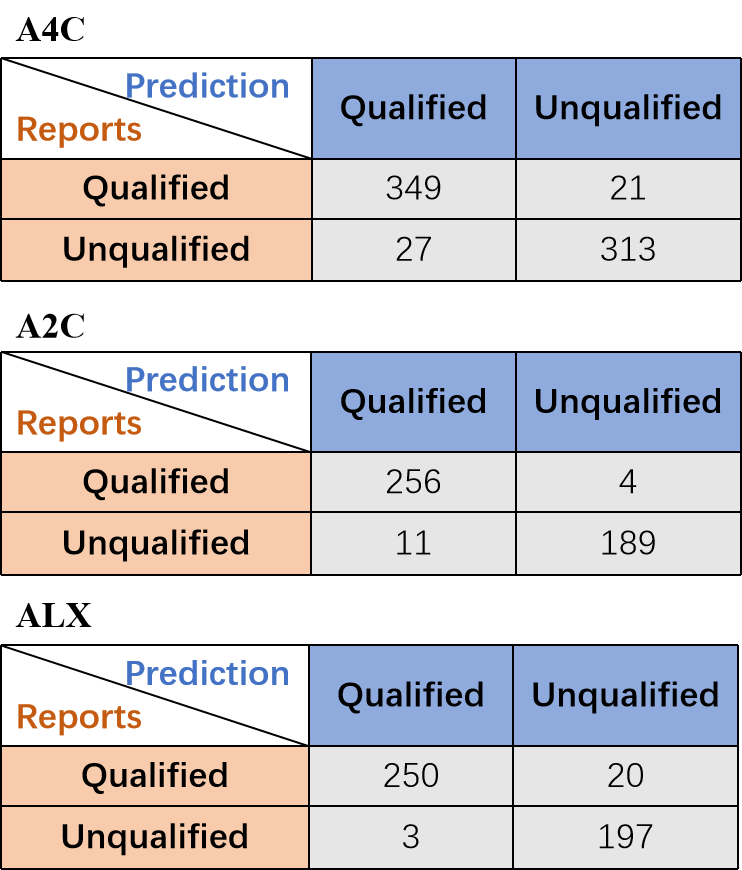


**Figure S2.** The Architecture for RWMAs Detection Model


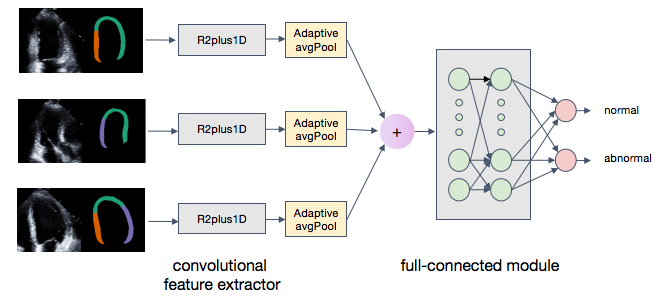


**Figure S3.** Spatiotemporal Video Augmentation

**
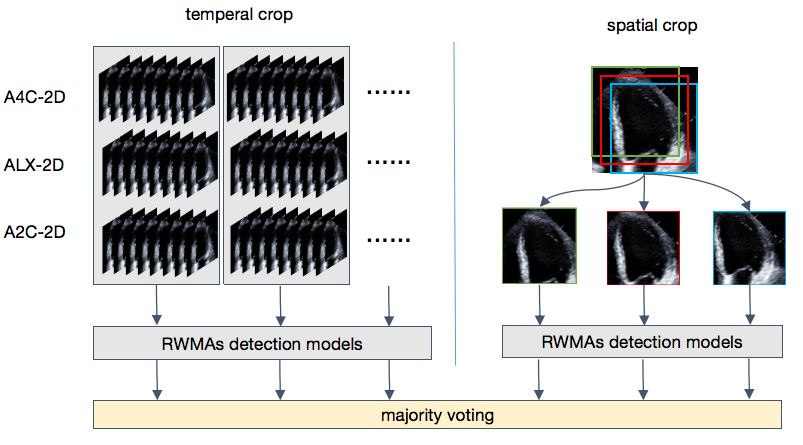
**

**Figure S4.** The Performance of View Classification Model

**
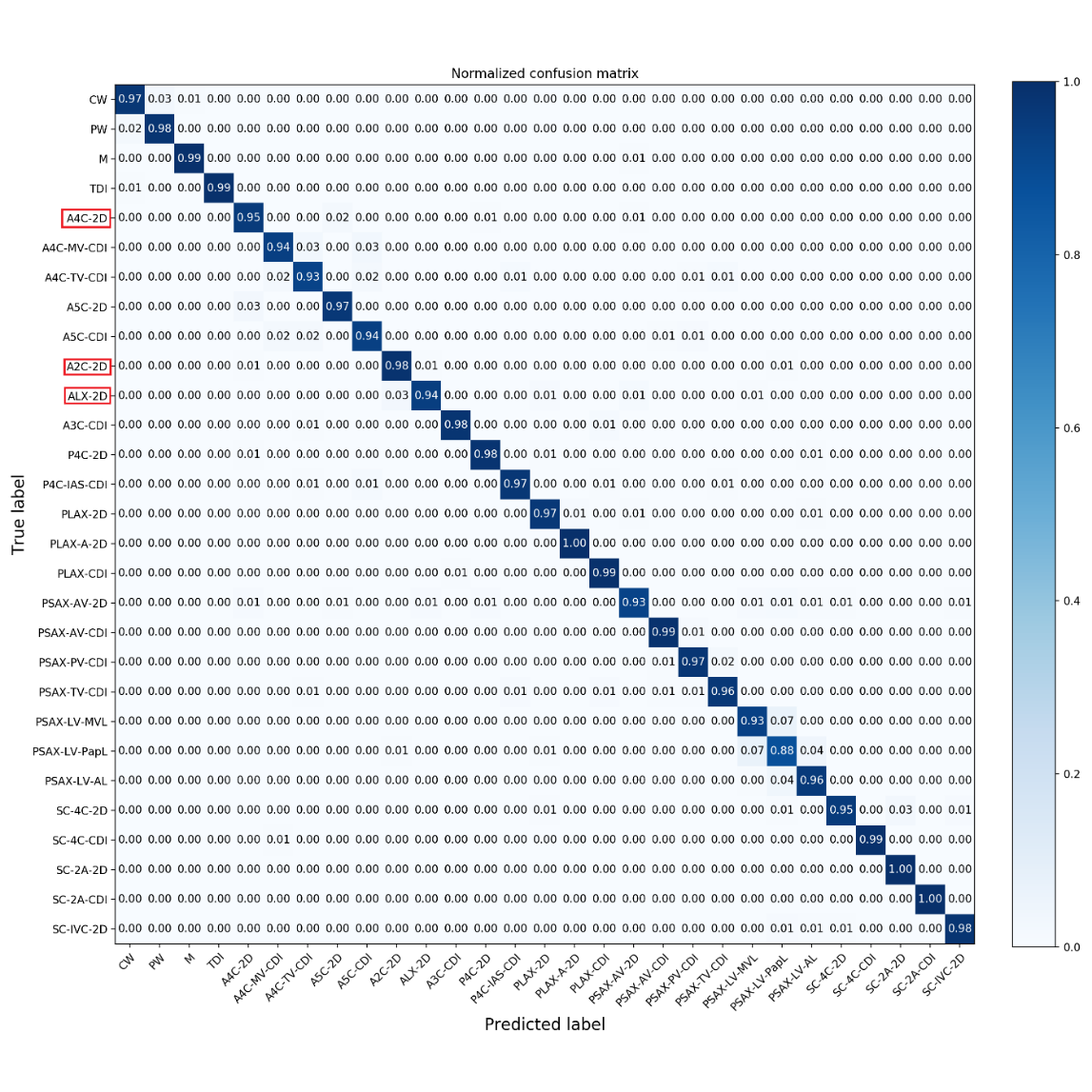
**

**Figure S5.** The Performance of Experts and Beginners in Detecting RWMAs with or without AI.

**
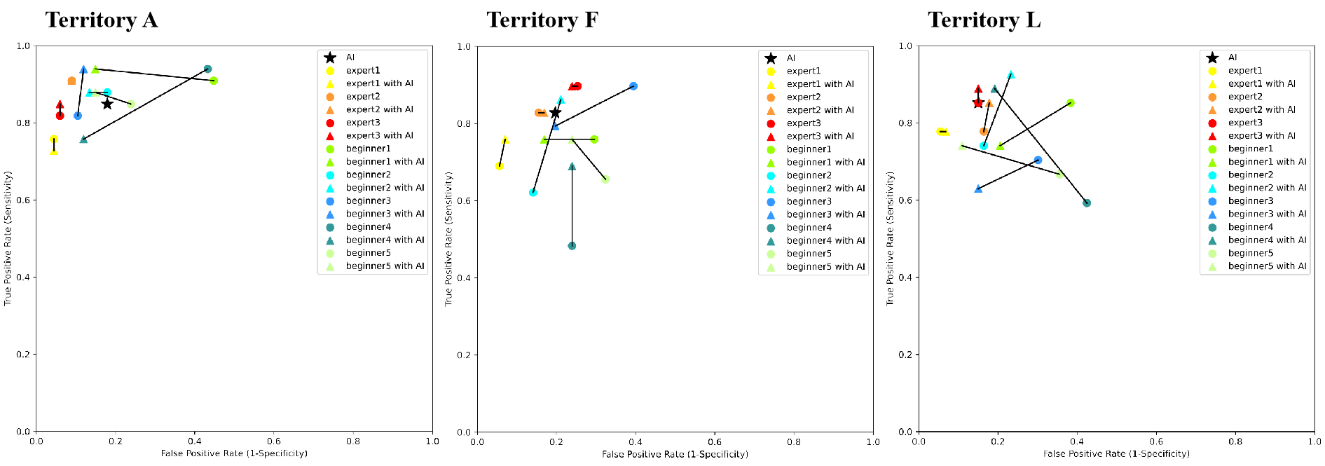
**

**Figure S6.**The Performance of the Automated Quantification Model in Internal and External Test Dataset

**
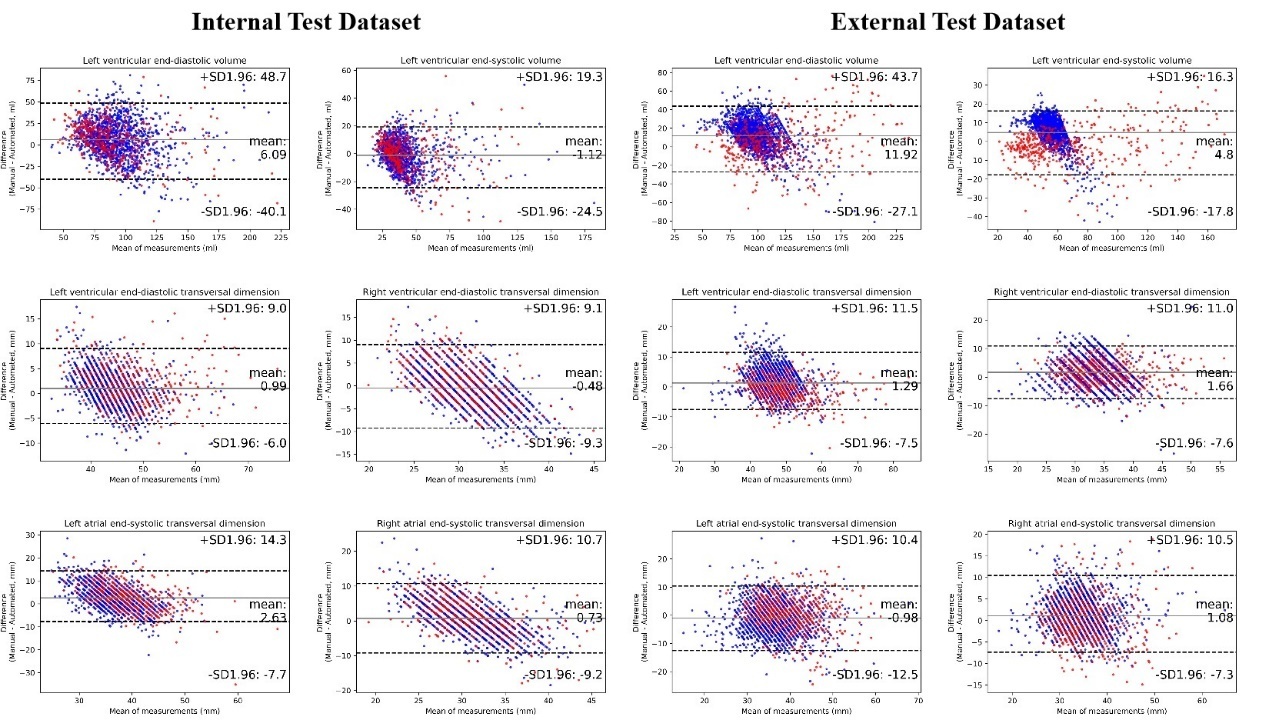
**

**Figure S7.** The Performance of RWMAs Detection Model and Automatic Quantification Model in the Unqualified Dataset


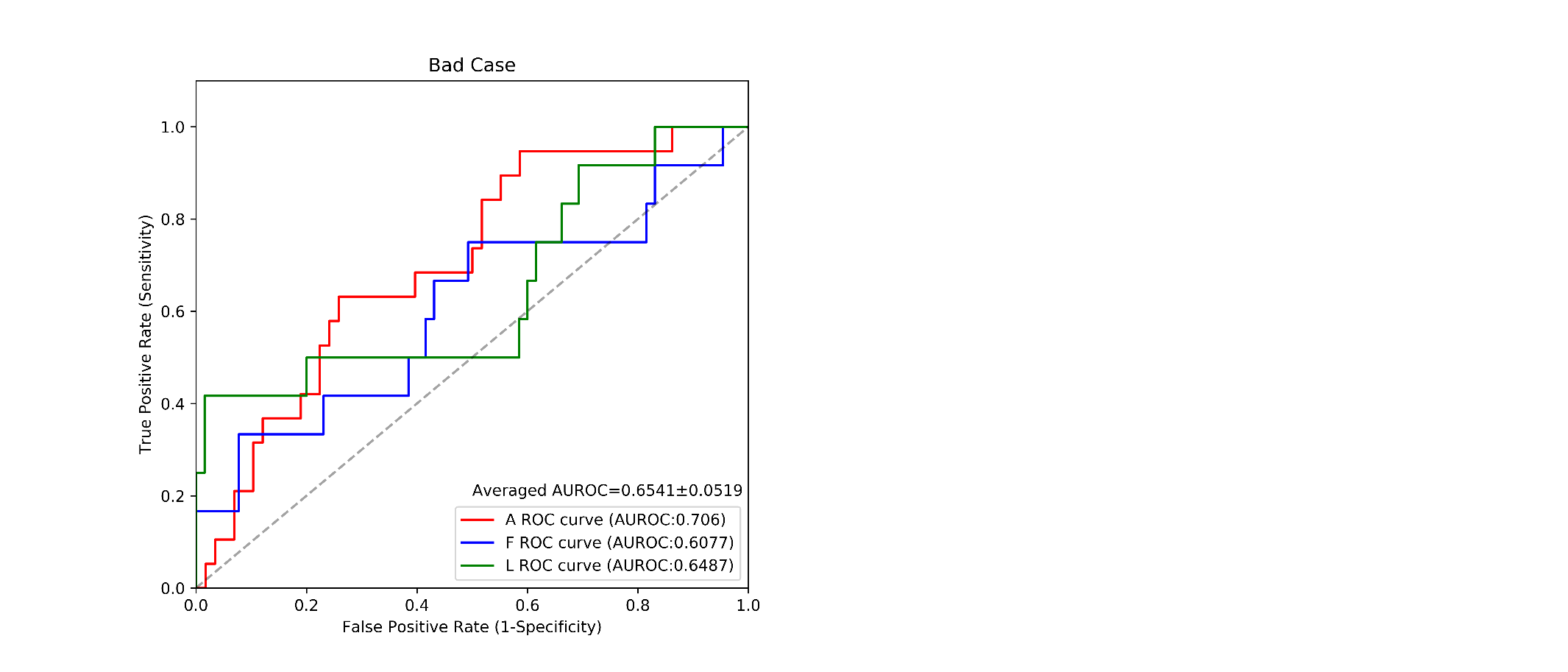

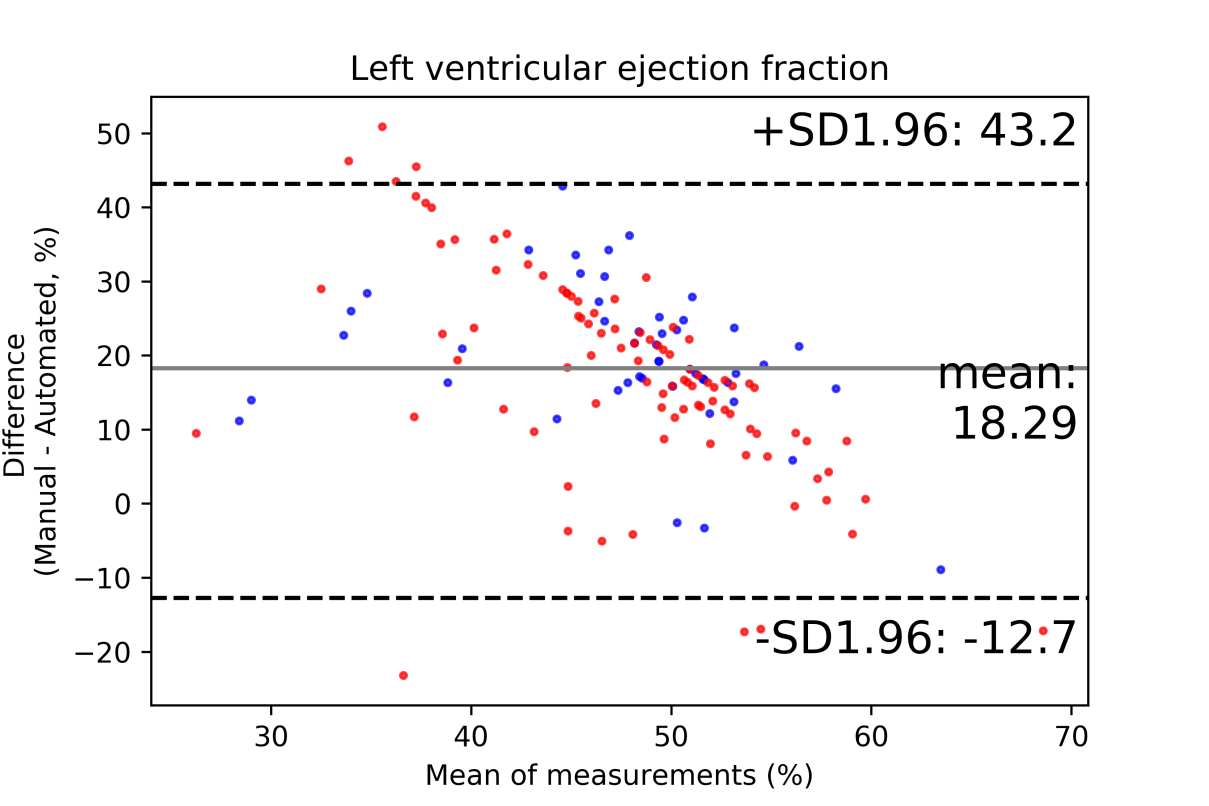


**Figure S8.** The distribution of age in correct and incorrect cases.


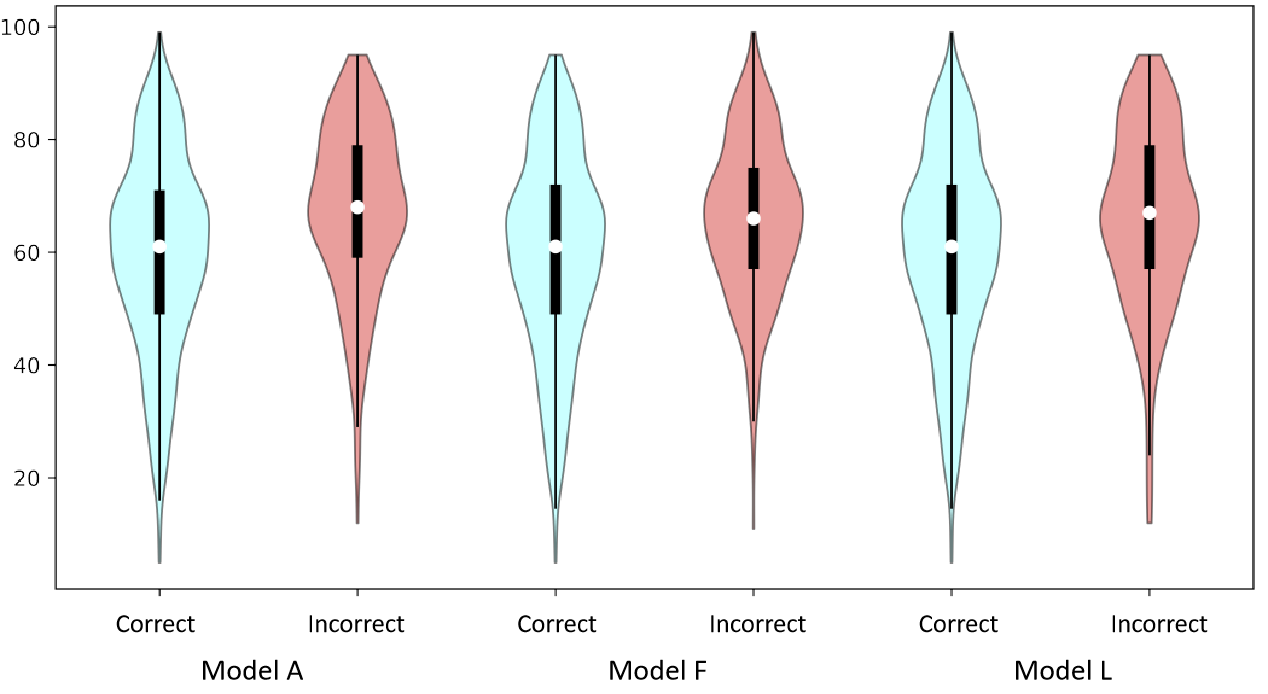

Supplement: Supplementary file 1 [file Data_Sheet_1.docx]
